# Defensive changes in maize leaves induced by feeding of Mediterranean corn borer larvae

**DOI:** 10.1186/s12870-017-0991-9

**Published:** 2017-02-15

**Authors:** Rogelio Santiago, Ana Cao, Ana Butrón, Ana López-Malvar, Víctor M. Rodríguez, Germán V. Sandoya, Rosa A. Malvar

**Affiliations:** 1 Universidad de Vigo, Agrobiología Ambiental Calidad de Suelos & Plantas UVIGO, Unidad Asociada MBG CSIC, Vigo, 36310 Spain; 2 Misión Biológica de Galicia CSIC, Apartado 28, Pontevedra, 36080 Spain; 3 The Genome Center and Department Plant Sciences, University of California, Davis - USDA-ARS. 1636 E. Alisal St., Salinas, CA 93905 USA; 4 Dept. Biología Vegetal & Ciencias Suelo, Facultad de Biología, Campus Lagoas Marcosende, Vigo, 36310 Spain

**Keywords:** Induced response, Maize antibiosis, Oral secretions, Cell wall hydroxycinnamates, DIMBOA

## Abstract

**Background:**

Plants can respond to insect attack via defense mechanisms that reduce insect performance. In this study, we examined the effects of several treatments applied to two maize genotypes (one resistant, one susceptible) on the subsequent growth and survival of *Sesamia nonagrioides* Lef. (Mediterranean corn borer, MCB) larvae. The treatments were infestation with MCB larvae, application of MCB regurgitant upon wounding, wounding alone, or exposure to methyl jasmonate, and they were applied at the V6–V8 stage of maize development. We also monitored changes in the concentrations of compounds known to be involved in constitutive resistance, such as cell wall-bound hydroxycinnamates and benzoxazinoids.

**Results:**

In both maize genotypes, the leaves of plants pre-infested with MCB larvae were less suitable for larval development than those from untreated plants. Application of MCB regurgitant upon wounding, and wounding itself, resulted in leaf tissues becoming less suitable for larval growth than those of pre-infested plants, suggesting that there could be herbivore-associated effector molecules that suppress some wounding responses. A single application of MCB regurgitant did not seem to mimic feeding by MCB larvae, although the results suggested that regurgitant deposited during feeding may have enhanced ferulates and diferulates synthesis in infested vs. control plants. Jasmonic acid may play a role in mediating the maize response to MCB attack, but it did not trigger hydroxycinnamate accumulation in the leaves to a level comparable to that induced by larval leaf feeding. The EP39 maize genotype showed an increase in leaf cell wall strength by increasing hemicellulose cross-linking in response to MCB attack, while induced defenses in the EP42 plants appeared to reflect a broader array of resistance mechanisms.

**Conclusions:**

The results indicated that leaf feeding by MCB larvae can increase leaf antibiosis against MCB in two maize genotypes with contrasting levels of resistance against this borer. Also, the larval regurgitant played a positive role in eliciting a defense response. We determined the effects of the plant response on larval growth, and detected defense compounds related to borer resistance.

**Electronic supplementary material:**

The online version of this article (doi:10.1186/s12870-017-0991-9) contains supplementary material, which is available to authorized users.

## Background

The Mediterranean corn borer (*Sesamia nonagrioides* Lef., MCB) is the main maize pest in the Mediterranean area [1, 2]. In Spain, the first generation of MCB larvae attack maize plants at an early stage of development and feed on leaves, and the second and further generations mainly feed on the pith [3]. Several studies have focused on identifying constitutive chemical compounds in maize that are involved in resistance to MCB, and the best candidates are benzoxazinoids and hydroxycinnamates [4, 5].

Benzoxazinoids are the most extensively studied cereal phytoalexins because they play a major role in the defense of cereals against insects, fungi, bacteria, and adventitious plant species [6]. In young maize plants, benzoxazinoids, especially DIMBOA (2,4-dihydroxy-7-methoxy-1,4-benzoxazin-3-one, the most abundant benzoxazinoid in maize), are considered to be the most important chemical factors in resistance to leaf-feeding insects, including corn borers such as the European corn borer, *Ostrinia nubilalis* Hübner*,* and MCB [6]. Benzoxazinoids are predominantly stored as glucosides in the cell vacuole. Tissue maceration by chewing herbivores results in the release of active aglycones by the action of endogenous β-glucosidases. Hydroxycinnamates, derived from the phenylpropanoid pathway, comprise another array of compounds that have been extensively studied in relation to their function in constitutive resistance to herbivores [7–9]. Grasses contain relatively high concentrations of ferulates (FAs) and *p*-coumarates (*p*CAs) linked to hemicellulose and lignin polymers. The FAs are attached by ester bonds to arabinose side chains of arabinoxylans and can be coupled by oxidative reactions to form dehydrodimers (diferulates, DFAs) that crosslink hemicellulose, or bind lignin monomers via ether bonds that cross-link hemicellulose with lignin. In cereals, *p*CAs are incorporated into cell walls where they are ester-linked to lignin monolignols, and are thought to function in transferring radicals during the polymerization of lignin [7, 10]. The constitutive contents of *p*CAs and FAs in cell walls and the degree of hemicellulose cross-linking by DFA bridges has been suggested to be a structural defense mechanism against insect damage. Several studies reported that differences in cell wall-bound hydroxycinnamate contents in grains, leaves, or stem tissues between resistant and susceptible genotypes of maize, wheat, and tall fescue were associated with contrasting levels of resistance to folivores and stem borers and with reduced insect performance [5, 8, 9, 11–13].

In addition to constitutive resistance, maize shows other responses induced by insect feeding. Khajuria et al. [14] described the up-regulation of various enzymes in the phenylpropanoid metabolic pathway in wheat after infestation by Hessian fly (*Mayetiola destructor* Say) that led to increased abundance of the phenolics 4-hydroxy-cinnamate and vanillin. Several studies have focused on the induction of benzoxazinoid accumulation in leaves by insect attack. In maize leaves, the transcript levels of *Bx1*, the first gene in the benzoxazinoid biosynthesis pathway, were shown to increase after caterpillar attack [15, 16]. In parallel, the benzoxazinoid content in maize leaves changed after infestation by specialist and generalist caterpillars; insect feeding led to significant increases in DIMBOA and HDMBOA-Glc (the methylated form of DIMBOA-Glc) and decreased levels of DIMBOA-Glc, and the younger leaves were more prone to benzoxazinoid induction than older ones [15, 17–19]. In relation to MCB attack, Rodriguez et al. [20] showed that maize stalks near the flowering stage responded to MCB attack by activating general plant defense mechanisms, including genes encoding jasmonic acid biosynthetic enzymes, proteinase inhibitors, defense-related transcription factors, and proteins involved in cell-wall reorganization. Concomitantly, after MCB attack, all genotypes showed no significant decreases in DIMBOA content, while some showed significant changes in the levels of specific hydroxycinnamate compounds that were probably involved in cell-wall stiffness. However, the induced response to MCB at earlier stages of maize development, when maize leaves are damaged by first-generation larvae, has not been studied, and there is no information about the induction of resistance-related metabolites at this stage of development.

Furthermore, little is known about insect and plant metabolites involved in eliciting the response to MCB attack and in perceiving and signaling damage caused by this lepidopteran species. Plants likely use two strategies to optimize their response to herbivore feeding: damaged-self recognition, and herbivore-associated molecular patterns (HAMPs)-based specific responses. However, HAMPs-triggered immunity can be partially suppressed by herbivore-associated effector molecules [21, 22]. HAMPs have been found in oral secretions such as regurgitant and those of salivary glands. These secretions have been shown to modulate specific defense responses to herbivores, either amplifying or suppressing direct and indirect defenses elicited by wounding [23–27]. HAMPs identified in the regurgitant or saliva of insect species include amino acid-fatty acid conjugates (FACs), enzymes such as β-glucosidase and glucose oxidase, fragments of ingested plant proteins, sulfated fatty acids, and cell wall fragments [27, 28]. In maize, the regurgitants of Lepidoptera species such as *Spodoptera littoralis* (Boisduval) and *Spodoptera exigua* Hübner were shown to induce plants to release volatile compounds and trigger direct responses [15, 29–31]. So far, no studies have determined presence of HAMPs in MCB regurgitant. Identification of such HAMPs is the first step to determine whether MCB regurgitant can promote maize defense responses and increase resistance. Similarly, although jasmonic acid has been proposed as an important molecule in mediating the maize response to herbivore attack, little is known about its role as a signaling molecule in the induced response to MCB [32]. Recently, methyl jasmonate (MeJA) was suggested to play an important role in maize defense signaling against Asian corn borer (*Ostrinia furnacalis*) attack [33].

The overall aim of this study was to expand our knowledge about the factors involved in triggering and signaling the maize response to first-generation MCB attack, to determine if constitutive resistance metabolites respond to first-generation MCB attack, and to assess whether the induced response increases leaf resistance to MCB. The specific objectives were as follows: 1) to determine whether the response to leaf feeding by MCB larvae increases leaf antibiosis against MCB in two maize genotypes with contrasting levels of resistance against MCB; 2) to determine if MCB regurgitant has a role in eliciting that response; 3) to ascertain if MeJA functions in mediating the maize response; and 4) to evaluate the effect of several treatments (feeding by MCB larvae, exposure to MeJA, and application of MCB regurgitant upon wounding) on metabolites that are known to be involved in constitutive resistance to MCB attack.

## Results

### Non-choice feeding bioassays

**Fig. 1:**
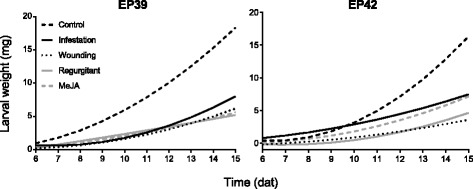
Mediterranean cron borer (MCB) larval growth in non-choice feeding bioassays. Regression curves of weight of the MCB larvae fed on leaves of EP39 and EP42 maize plants pre-infested with MCB larvae, wounded, treated with MCB regurgitant upon wounding, exposed to methyl jasmonate (MeJA) or untreated (control) over time. Bioassays were initiated at 2 days after treatment (dat)

To evaluate if feeding by MCB increases the antibiosis of leaves in young plants, MCB larvae were reared for 13 days on excised leaves from maize plants previously infested with MCB larvae (48 h before), and larval weight and survival were monitored. The growth and survival on leaves from plants in other treatments (wounding, application of regurgitant upon wounding, and exposure to MeJA) and untreated plants (control) were monitored at the same time. The regression curves of larval weights with time (growth curves) showed that larvae grew better on leaves from untreated plants than on those from treated plants regardless of the maize genotype (Fig. 1, Additional file 1: Table S1). However, the differences among treatments varied between the two maize genotypes. The antibiosis levels of the leaves from EP39 and EP42 plants pre-infested with MCB larvae were similar at the early stage of the bioassay (6 days after treatment; dat) (*p* > 0.05) (Table 1). Leaves of EP42 had a stronger antibiotic effect on larval weight than those of EP39 at 15 days after wounding, but the opposite effect was observed when leaves were treated with MeJA. The responses to treatments differed slightly between the two maize genotypes; differences among treatments in EP39 became significant at 8 dat, while differences in EP42 became significant at 10 dat. The effect of MeJA treatment was similar to that of infestation treatment in EP42 at 15 dat, while the effect of MeJA treatment was similar to those of the wounding treatment and the regurgitant treatment in EP39. The wounding treatment and the regurgitant treatment negatively affected larval development on both maize genotypes at 15 dat (Table 1).

**Table 1:** Mediterranean corn borer larval weights in non-choice feeding bioassays

Genotype	Treatment	Days of feeding^a^									
		6 dat		8 dat		10 dat		13 dat		15 dat	
EP39	Control	3.52	a	5.21	a	7.97	a	15.51	a	20.46	a
	Infestation	3.09	a	3.71	ab	4.02	bc	7.01	c	10.49	b
	Wounding	2.71	a	2.90	b	3.97	bc	6.66	cd	8.47	bcd
	Regurgitant	2.68	a	3.51	ab	5.16	b	6.30	cde	7.78	cde
	MeJA	2.46	a	3.46	b	4.00	bc	4.54	e	7.16	de
EP42	Control	2.68	a	4.29	ab	5.58	b	11.05	b	18.96	a
	Infestation	3.24	a	4.28	ab	5.37	b	7.66	c	10.04	b
	Wounding	2.33	a	2.89	b	3.07	c	4.88	de	5.90	e
	Regurgitant	2.29	a	2.48	b	2.93	c	4.88	de	7.22	de
	MeJA	2.53	a	3.22	b	4.32	bc	6.82	cd	9.68	bc

Least square (LS) mean values of larval weight (mg) of MCB larvae fed on leaves from maize plants pre-infested with MCB larvae, wounded, treated with MCB regurgitant upon wounding, exposed to methyl jasmonate (MeJA) or untreated (control). Bioassays were initiated 2 days after treatment (dat)

^a^Within each column, different letters indicate significant differences (*p* < 0.05)

The larval survival curves differed significantly among treatments when larvae were fed with leaves from EP42 (log-rank test, *p* = 0.02), but not when they were fed with leaves from EP39 (log-rank test, *p* = 0.34) (Table 2). Wounding was the only treatment that significantly reduced the survival of larvae fed with EP42 leaves, compared with control plants (*p* < 0.05) (Table 2).

**Table 2:** Mediterranean corn borer larval survival in non-choice feeding bioassays

Treatment	EP39		EP42	
Control	−6.94	a	−4.16	bc
Infestation	2.88	a	−1.92	abc
Wounding	0.87	a	7.97	a
Regurgitant	−0.09	a	5.87	ab
MeJA	3.28	a	−7.77	c

Values of log-rank statistic for testing homogeneity of survival distribution of MCB when larvae were reared on previously infested, wounded, treated with regurgitant upon wounding, treated with methyl jasmonate (MeJA) and untreated (control) plants. Positive values of log-rank statistic indicate that number of dead larvae is greater than that expected under the null hypothesis of equivalent survival distributions

^a^Log-rank statistics followed by the same letter in the same row indicate that survival curves were homogeneous (*p* > 0.05)

### Cell wall bound hydroxycinnamates and DIMBOA concentrations in maize leaves

**Fig. 2:**
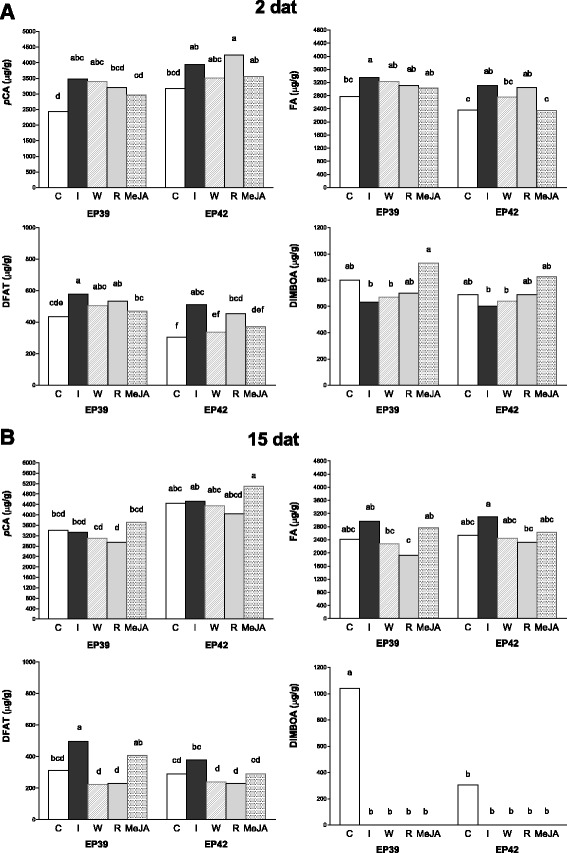
Cell wall-bound hydroxycinnamate and 2,4-dihydroxy-7-methoxy-1,4-benzoxazin-3-one (DIMBOA) concentrations in maize leaves. Mean concentration of cell wall bound hydroxycinnamates and DIMBOA (μg/g dry weight) in the leaves of maize inbreds EP39 and EP42 at 2 (**a**) and 15 (**b**) days after (dat) infestation with Mediterranean corn borer (MCB) larvae (I), wounding (W), wounding + MCB regurgitant application (R), methyl jasmonate exposure (MeJA), and no treatment (C). *p*CA, p-coumarate; FA, ferulate; DFAT, total diferulates

Next, we determined whether leaf feeding by MCB larvae, wounding, wounding + MCB regurgitant, or exposure to MeJA affected cell wall-bound hydroxycinnamates and DIMBOA concentrations in the leaves. The concentrations of *p*CA, FAs, total DFAs (DFAT), and DIMBOA in the leaves of treated and control plants were compared at 2 and 15 dat. Leaf feeding by MCB larvae resulted in significantly increased contents of cell wall-bound FAs and DFAT in the leaves of EP39 and EP42 at 2 dat (*p* < 0.05) (compared with control), while the increase in *p*CAs was only significant in EP39 leaves (Fig. 2a, Additional file 1: Table S2). At 15 dat, the DFAT concentration in leaves infested by MCB was higher than that in leaves of untreated plants, but the difference was only significant for EP39 (Fig. 2b, Additional file 1: Table S2). Interestingly, application of MCB regurgitant to wounded leaves produced short-term increases (2 dat) in the level of cell wall-bound hydroxycinnamates, similar to those reported after leaf feeding by MCB larvae. In EP42, regurgitant application resulted in higher *p*CAs, FAs, and DFAT contents in leaves than did wounding alone, although this difference was only significant for DFAT contents (*p* < 0.05) (Fig. 2a, Additional file 1: Table S2). In the long term, wounding and the application of regurgitant had similar effects on hydroxycinnamates content, and those effects were the opposite of those induced by larval feeding (Fig. 2b, Additional file 1: Table S2). Exposure to MeJA produced slight increases (not significant) in cell wall-bound hydroxycinnamates contents in the leaves over time (Fig. 2a and b).

We detected DIMBOA in treated and control leaves at 2 dat, and only the MeJA treatment increased the DIMBOA concentration to a level higher than that in the control. The wounding, wounding + regurgitant, and infestation treatments resulted in decreased DIMBOA contents (*p* > 0.05). This opposite changes in DIMBOA contents between infested/wounded plants and MeJA-treated plants resulted in significantly different concentrations of DIMBOA in EP39 plants (*p* < 0.05) (Fig. 2a, Additional file 1: Table S2). No DIMBOA was detected in the treated leaves at 15 dat, and the DIMBOA content in leaves of untreated plants was 3-fold higher in EP39 (1041.72 μg/g) than in EP42 (303.65 μg/g) (*p* < 0.05) (Fig. 2b, Additional file 1: Table S2).

## Discussion

Insect attack induces defense mechanisms in plants that can reduce the fitness of the herbivorous insect. In maize, negative effects on the growth of larvae fed on leaves previously exposed to insect feeding or insect elicitors has been reported for noctuid species such as *S. littoralis* and *Spodoptera frugiperda* [15, 29, 34], and for the crambid *O. nubilalis* [26]. In this study, the effect of leaf feeding by MCB on the suitability of leaves for MCB growth was assessed for 13 days (from 2 dat to 15 dat) in two maize genotypes. We found that MCB larvae grow slower on leaves from previously infested plants, regardless of the maize genotype. The antibiotic effect triggered in leaves by insect feeding was only relevant in the long term, because differences in larval weights between those reared on untreated and previously infested plants became significant only after 8 days of feeding. These results highlight the relevance of performing long-term bioassays, because the antibiosis effect could be exponential rather than linear, and/or plant responses to short and long-term infestation could differ due to continuous cross-talk between the plant and the larvae. The plant response triggered by the first contact with the insect may differ from that induced later, so that the metabolic arsenal of the larvae may be modified as a counter-response to plant induction. The EP39 response to infestation seemed to contribute earlier than that of EP42 to leaf antibiosis. However, at the end of bioassay, the antibiosis levels of EP42 and EP39 leaves from infested plants were similar. Studies on other chewing herbivores have shown that leaf feeding can increase plant resistance, which can be measured as reduced larval growth. However, this effect depends on the insect species, plant genotype, and the interval between priming and evaluating antibiosis [35, 36]. We conclude that leaf feeding increased resistance to MCB in two different maize genotypes, but the response to MCB attack was not immediate.

The application of MCB regurgitant upon wounding did not significantly increase the antibiosis of maize leaves more than mechanical wounding alone, because the plant responses elicited by MCB regurgitant + wounding and wounding alone retarded larval growth to the same extent. Therefore, MCB regurgitant does not appear to play an important role in eliciting direct defenses. Alternatively, the procedure used to apply the regurgitant may need improvement, because the regurgitant was applied once, rather than multiple times over time, which would more closely mimic possible regurgitant secretion during feeding. Also, because the response of maize plants to insect regurgitant depends on plant age, time of exposure, and maize genotype [31, 34, 37], we cannot rule out that regurgitant from MCB larvae could play a crucial role in inducing maize defense mechanisms at other plant stages and/or in maize genotypes different from those studied here. Regurgitant from pests such as *S. exigua, S. littoralis,* and *Mythimna separata* (Walker) was shown to induce defense-related genes and jasmonic acid accumulation [15, 29–31, 38], but regurgitant from other corn borers present in Europe, such as the European corn borer (*O. nubilalis*), did not elicit direct defenses in maize leaves [26]. In this last case, it was the saliva from *O. nubilalis*, and not the regurgitant, that was the critical component for inducing direct plant defenses in maize [26].

The results of the bioassay indicated that HAMPs were not present in MCB regurgitant, but suggested that herbivore-associated effector molecules could be present in some other MCB secretions because the wounding and MCB regurgitant + wounding treatments resulted in lower larvae growth than did actual herbivory. This could indicate that MCB larvae partially counteract plants’ responses to mechanical damage, making the infested leaves less harmful for consumption than mechanically wounded leaves. Salivary and frass effectors in other Lepidoptera species that disrupt plant defense signaling are currently being studied [27, 39–41]. In addition, we speculate that the possible effectors present in MCB frass or saliva could suppress some of the maize defenses against MCB attack mediated by jasmonic acid, as reported for the maize–*S. frugiperda* interaction [40]. However, this partial suppression of defenses would depend on the maize genotype, because MeJA and infestation treatments had similar effects on the antibiosis of EP42 leaves, but MeJA and wounding treatments had similar effects on the antibiosis of EP39 leaves. In EP39, the combination of MeJA + wounding reduced larval growth more than did the infestation treatment.

After investigating the effects of plant treatments (infestation, MCB regurgitant + wounding, and exposure to MeJA) on MCB growth and survival, we determined whether these treatments induced the accumulation of resistance-related compounds (DIMBOA and cell wall-bound hydroxycinnamates) in leaves. At 2 dat, maize plants in all treatments and both genotypes had DIMBOA levels ranging from 600 to 932 μg/g dry weight. However, at 15 dat, no DIMBOA was detected in treated plants; while the concentration in untreated EP39 and EP42 plants was 1042 and 304 μg/g dry weight, respectively. Several studies have reported that HDMBOA-Glc was strongly induced in maize soon (24–48 h) after herbivory or jasmonic acid elicitation, while DIMBOA-Glc levels decreased [17, 18, 39]. We propose that longer term (15 dat) tissue disruption and exposure to MeJA could result in total conversion of DIMBOA-Glc to HDMBOA-Glc to better deter herbivory by specialist insects, because HDMBOA, unlike DIMBOA, cannot be detoxified by insects via glycosylation [19]. At this stage of development and using the current methodology, the only peak at quantifiable concentrations was that corresponding to DIMBOA. Further in-depth research using precise analytical procedures needs to be carried out to identify and quantify the presence of derivates at lower concentrations.

We also investigated whether induced cell wall fortification mediated by hydroxycinnamates occurs in maize leaves after MCB attack, and tested whether MCB regurgitant and jasmonic acid play roles in elicitation and signaling. Maize plants infested with MCB larvae showed increased cell wall FAs and DFAT contents in leaves at all sampling times, suggesting that leaf feeding by MCB larvae modified the leaf cell wall structure by increasing feruloylation of arabinoxylan chains and favoring hemicellulose cross-linking. Changes in hydroxycinnamate contents in maize stalk after MCB attack have been reported previously [20]. In the short term, the effects of MCB regurgitant + wounding on FAs and DFAT contents were similar to the effect of larval feeding, but a significant effect of MCB regurgitant + wounding on FA and DFAT contents was detected at 15 dat. Therefore, the continuous contact of the maize plant with regurgitant elicitors during larval feeding may have a role in maintaining higher rates of FAs and DFAT synthesis in infested vs. control plants. Unlike regurgitant + wounding and infestation treatments, MeJA and wounding treatments did not cause a significant difference in DFAT compared with that in the control at 2 dat. This result suggested that the response mediated by jasmonic acid signaling or mechanical damage cannot solely explain the changes in FAs and DFAT contents in the infested plants. Cell wall *p*CAs were induced after infestation but, unlike FAs and DFAT, the *p*CAs levels were similar in infested and control plants at 15 dat. Therefore, although cell wall *p*CAs are related to lignin deposition and their abundance is positively correlated with lignin content in several plant species [7, 9, 42], they did not seem to participate in long-term leaf cell wall fortification in maize induced by MCB attack.

Increased feruloylation and polymer cross-linking by insect feeding could reinforce the leaf cell wall. This reinforcement is expected to hinder the activity of cell wall-degrading enzymes and affect the accessibility and digestibility of plant tissues, and hence, larval performance [8, 43]. Nevertheless, after infestation, the significant difference in hydroxycinnamate contents between genotypes (higher in EP39 than in EP42) at 15 dat was not associated with a significant difference in antibiosis against MCB. Therefore, other mechanisms induced by insect attack, besides hydroxycinnamate induction and DIMBOA metabolism, could be involved in reducing insect performance on leaves of EP42.

## Conclusions

In both maize genotypes, the leaf responses to MCB infestation retarded the growth of MCB larvae but barely affected larval survival. In maize, MCB regurgitant could play a role in inducing DFAT accumulation, but herbivore-associated effector molecules that suppress some responses triggered by wounding could also be involved in the maize–MCB interaction. In the EP39 maize line, the plants strengthened their leaf cell walls through incorporating hydroxycinnamates and increasing hemicellulose cross-linking in response to MCB attack, while the induced defenses in the EP42 line appeared to reflect a wider array of resistance mechanisms.

## Methods

### Insect rearing and regurgitant collection

The MCB larvae were obtained from the colony maintained at the insectary of the Misión Biológica de Galicia-CSIC (Spanish National Research Council). Second instar larvae (weight, 1–2 mg and approximately 5 mm body size) were fed on a maize-based artificial diet and then starved for 24 h before artificial infestation, growth, and survival bioassays. The MCB regurgitant was collected from 4^th^–5^th^ instar larvae previously fed on maize stem tissue for at least 48 h. Larvae were chilled on ice and immobilized. As larvae returned to room temperature they were squeezed until the regurgitant was expelled. Regurgitant from 50 larvae (300 μl approx.) was mixed with 150 μl 0.1 M phosphate buffer solution (PBS) and frozen. This mixture was applied to plants immediately after wounding as described below.

### Plant materials and treatment applications

Two different maize inbred lines (EP39 and EP42, both European flint lines) were used in this study. In previous studies, these two lines showed different gene ontology (GO) categories involved in the response to stem tunneling by MCB larvae [20], contrasting resistance to stem borer, and different cell wall hydroxycinnamate composition of pith tissues [3, 5, 44–46]. Maize plants were individually grown in pots in greenhouse conditions and arranged in a split-plot design, where treatments were assigned to the main plots and maize genotypes were assigned to sub-plots. Twenty plants for each treatment–genotype combination were planted. Five treatments were applied to maize plants at the V6–V8 stage (six to eight fully expanded leaves): infestation with MCB larvae, wounding, wounding + MCB regurgitant; exposure to MeJA; and control (untreated plants). In the infestation treatment, three 2^nd^ instar larvae were placed in the whorl of the maize plants and were allowed to feed freely. The pots were protected with nets to avoid larvae dispersion. In the wounding + MCB regurgitant treatment, three leaf wounds (scratches) of 5 × 5 mm were made with a scalpel on each plant, and 5 μl regurgitant mixture was applied to each wound using a micropipette. The total amount of regurgitant mixture applied to each plant was 15 μl (containing regurgitant from approximately 5 larvae). In the wounding treatment, wounding was conducted as described above and 5 μl PBS solution (15 μl PBS per plant) was applied to each wound. In a fourth treatment, plants were exposed to exogenous MeJA, a known elicitor of the defense response, by placing a cotton tip soaked with 100 μM MeJA dissolved in ethanol (10% v/v) in a leaf axil. Leaves from different plants of each treatment–genotype combination were collected for the non-choice feeding bioassay with MCB larvae, biochemical analyses, and RNA quantification.

### Non-choice feeding bioassays

The non-choice feeding assay was used to assess the effects of various plant treatments on the subsequent development and survival of MCB larvae reared on leaves from pre-conditioned plants. Two days after treatments were applied, 2^nd^ instar MCB larvae were initially weighed and placed in multi-well plates on fresh leaf discs. Twenty larvae per treatment and genotype were tested in duplicate (i.e., *n* = 80 larvae per treatment) and were maintained in a growth chamber under controlled temperature and humidity conditions (22 °C, 80% RH) under a 16 L:8D photoperiod. Every 2–3 days, new fresh leaf discs were provided to the larvae. Larval weights and data related to dead and missing larvae were recorded at 6, 8, 10, 13, and 15 dat.

A repeated-measure analysis was performed to test differences for larval weights using the PROC MIXED procedure of SAS software (SAS Institute, Inc., Cary, NC, USA) [47, 48]. Initial larval weight was included as a covariate, genotype was set as a random factor, and a first-order autoregressive covariance structure (AR-1) was chosen in the within-subject correlation. The initial larval weight was subtracted from the larval weight at each time point in each treatment and genotype, and values shown are least square (LS) means. Linear and quadratic coefficients for the regression of larval weight over time were obtained for each treatment–genotype combination. Within each genotype, larval growth curves were compared between pairs of treatments by making orthogonal contrasts between the two treatments’ regression parameters (intercept, linear, and quadratic components) (*p* ≤ 0.05) [48, 49]. The PROC LIFETEST procedure of SAS was used to test differences in larval survival among treatments within the same genotype using the Kaplan–Meier method [47]. The death of larvae was an important event, and numbers of missing and alive larvae at the end of the bioassay were recorded. The homogeneity of survival distribution was tested using the log-rank test (*p* ≤ 0.05).

### Quantification of cell wall bound hydroxycinnamates and benzoxazinoids

For each genotype–treatment combination, leaves from four and two plants were collected at 2 and 15 days after treatment (dat), respectively, frozen (−20 °C), and then lyophilized. Cell wall-bound hydroxycinnamates were extracted from 500-mg ground leaf samples (two replicates), as described previously [46]. Briefly, samples were extracted in 80% methanol for 1 h and then centrifuged. The pellet was shaken in 2 N NaOH under nitrogen for 4 h. Digested samples were neutralized with 6 N HCl and the pH was lowered to 2.0. After centrifugation, the supernatant was collected and the pellet was washed twice with distilled water. Supernatants were pooled and then extracted twice with ethyl acetate. Collected organic fractions were combined and reduced to dryness using a Savant Speed Vac (Savant Instruments, Holbrook, NY, USA). The final extract was dissolved in 1.5 ml methanol, filtered through a 20-μm pore tetrafluoroethylene filter, and stored at −20 °C. For DIMBOA analysis, 100-mg ground leaf samples were extracted in 5 ml HPLC-grade methanol and 50 μl acetic acid. The mixture was vortexed and then incubated in a sonicator water bath for 60 min at 60 °C. The supernatant (0.5 ml) was combined with 0.5 ml distilled water in a microcentrifuge tube, vortexed, and centrifuged for 5 min at 1000 × g. The supernatants were transferred into vials and stored.

The HPLC analyses were performed using a 2690 Waters Separation Module (Waters, Milford, MA, USA) equipped with a 996 Photodiode Array Detector (Waters) with a YMC ODS-AM (Waters) narrow bore column (100 × 2 mm i.d.; 3 μm particle size). For elution, the mobile phase consisted of acetonitrile (solvent A) and trifluoroacetic acid (0.05%) in water (solvent B) delivered under the following gradient conditions: initial A:B ratio of 10:90, changing to 30:70 in 3.5 min, then to 32:68 in 6.5 min, then to 100:0 in 4 min, then isocratic elution with 100:0 for 4.5 min, and finally returning to initial conditions after 3 min. The flow rate was 0.3 ml/min. The sample injection volume was 4 μl, and the elution profiles were monitored by UV absorbance at 325 and 254 nm. Retention times and UV spectra were compared with those of freshly prepared standard solutions (*p*CA, FA, 5-5-DFA, and DIMBOA). *P*-Coumaric and ferulic acids obtained from Sigma (St. Louis, MO, USA), 5-5′ DFA was synthesized by the group of N. Towers (University of British Columbia, Vancouver, Canada), and DIMBOA was provided by the group of C. Souto (University of Vigo, Pontevedra, Spain). The UV spectra of other DFAs were compared with previously published spectra [50]. We identified and quantified four isomers of DFA: 5-5′DFA, 8-5′DFA (sum of 8-5′-non-cyclic and 8-5′-benzofuran forms), and 8-o-4′DFA. The sum of these isomers represented the total DFA (DFAT) content. The results are expressed as μg/g dry weight. Combined analyses of variance (ANOVA) for each compound concentration at each sampling time (2 and 15 dat) were performed with the GLM procedure of SAS, and all factors, except replicates, were considered fixed. Differences in compound contents between genotypes within each treatment were tested by comparing LS means (*p* ≤ 0.05).

## Additional files

Additional file 1: Supplementary Tables. **Table S1.** Estimates of parameters for regression of MCB larval weight over time; **Table S2.** Cell wall-bound hydroxycinnamates and 2,4-dihydroxy-7-methoxy-1,4-benzoxazin-3-one (DIMBOA) concentrations in maize leaves. (DOCX 23 kb)
Additional file 2:﻿ Bioassays raw data. (XLSX 33 kb)

## Abbreviations

Dat: days after treatment
DFA: Diferulates
DFAT: Total diferulates
DIMBOA: 2,4-dihydroxy-7-methoxy-1,4-benzoxazin-3-one
FA: Ferulates
HAMPs: Herbivore-associated molecular patterns
MCB: Mediterranean corn borer (*Sesamia nonagrioides*)
MeJA: Methyl jasmonate
*p*CA: *p*-coumarates
