# Supplementary material for: Defensive changes in maize leaves induced by feeding of Mediterranean corn borer larvae
Source: BMC Plant Biol. 2017 Feb 15;17:44. doi: 10.1186/s12870-017-0991-9 (PMC5312564; doi:10.1186/s12870-017-0991-9)
Supplement: Additional file 1: — Supplementary Tables. Table S1. Estimates of parameters for regression of MCB larval weight over time; Table S2. Cell wall-bound hydroxycinnamates and 2,4-dihydroxy-7-methoxy-1,4-benzoxazin-3-one (DIMBOA) concentrations in maize leaves. (DOCX 23 kb) [file 12870_2017_991_MOESM1_ESM.docx]

**Table S1.** Estimates of parameters for regression of MCB larval weight over time.

|  | **EP39** | | |  | **EP42** | | |
| --- | --- | --- | --- | --- | --- | --- | --- |
| **Treatment** | **Intercept** | **Linear** | **Quadratic** |  | **Intercept** | **Linear** | **Quadratic** |
| Control | 0.37 ± 1.35 ab | -0.4 ± 0.33 ab | 0.14 ± 0.02 ** a |  | 4.53 ± 1.56 ** a | -1.90 ± 0.37 ** b | 0.22 ± 0.02 ** a |
| Infestation | 3.02 ± 1.51 * a | -1.02 ± 0.38 ** b | 0.11 ± 0.02 ** ab |  | 0.11 ± 1.51 b | -0.01 ± 0.37 a | 0.04 ± 0.02 * b |
| Wounding | 0.85 ± 1.46 ab | -0.41 ± 0.36 ab | 0.06 ± 0.02 ** bc |  | -0.31 ± 1.74 b | -0.09 ± 0.43 a | 0.03 ± 0.03 b |
| Regurgitant | -1.60 ± 1.48 b | 0.45 ± 0.37 a | 0.01 ± 0.02 c |  | 1.59 ± 1.76 ab | -0.74 ± 0.42 a | 0.08 ± 0.02 ** b |
| MeJA | -0.02 ± 1.39 ab | -0.10 ± 0.35 ab | 0.03 ± 0.02 c |  | 0.71 ± 1.47 ab | -0.44 ± 0.35 a | 0.07 ± 0.02 ** b |

Intercepts and linear and quadratic coefficients (± SE) of the regression of weight of the MCB larvae fed on leaves from maize plants pre-infested with MCB larvae, wounded, treated with MCB regurgitant upon wounding, exposed to methyl jasmonate (MeJA) or untreated (control) over time.

^a^ Coefficients followed by one or two asterisks are significantly different from zero (*p* < 0.05 and *p* < 0.01, respectively).

^b^ Coefficients within a column followed by the same letter are homogeneous (*p* < 0.05).

**Table S2.** Cell wall-bound hydroxycinnamates and 2,4-dihydroxy-7-methoxy-1,4-benzoxazin-3-one (DIMBOA) concentrations in maize leaves.

|  |  | **2 dat^b^** | | | | | | | |  | **15 dat** | | | | | | | |
| --- | --- | --- | --- | --- | --- | --- | --- | --- | --- | --- | --- | --- | --- | --- | --- | --- | --- | --- |
| **Genotype** | **Treatment** | ***p*CA^a^** |  | **FA** |  | **DFAT** |  | **DIMBOA** |  |  | ***p*CA** |  | **FA** |  | **DFAT** |  | **DIMBOA** |  |
| EP39 | Control | 2432.41 | d | 2768.15 | bc | 434.74 | cde | 798.86 | ab |  | 3404.37 | bcd | 2420.59 | abc | 312.55 | bcd | 1041.72 | a |
|  | Infestation | 3478.35 | abc | 3357.90 | a | 576.99 | a | 633.67 | b |  | 3331.82 | bcd | 2967.47 | ab | 497.11 | a | 0.00 | b |
|  | Wounding | 3387.96 | abc | 3238.34 | ab | 506.04 | abc | 672.25 | b |  | 3090.63 | cd | 2281.65 | bc | 223.03 | d | 0.00 | b |
|  | Regurgitant | 3201.92 | bcd | 3106.17 | ab | 534.11 | ab | 699.60 | ab |  | 2952.11 | d | 1930.77 | c | 229.59 | d | 0.00 | b |
|  | MeJA | 2957.12 | cd | 3026.28 | ab | 471.62 | bc | 931.57 | a |  | 3710.70 | bcd | 2761.63 | ab | 406.12 | ab | 0.00 | b |
|  |  |  |  |  |  |  |  |  |  |  |  |  |  |  |  |  |  |  |
| EP42 | Control | 3172.43 | bcd | 2369.48 | c | 306.11 | f | 690.75 | ab |  | 4447.06 | abc | 2536.93 | abc | 288.45 | cd | 303.65 | b |
|  | Infestation | 3943.50 | ab | 3104.58 | ab | 510.93 | abc | 600.05 | b |  | 4518.91 | ab | 3105.28 | a | 379.13 | bc | 0.00 | b |
|  | Wounding | 3501.39 | abc | 2758.71 | bc | 337.73 | ef | 639.11 | b |  | 4357.48 | abc | 2447.53 | abc | 240.08 | d | 0.00 | b |
|  | Regurgitant | 4249.79 | a | 3046.67 | ab | 455.15 | bcd | 690.21 | ab |  | 4038.64 | abcd | 2316.68 | bc | 229.07 | d | 0.00 | b |
|  | MeJA | 3561.73 | ab | 2345.97 | c | 370.35 | def | 824.93 | ab |  | 5100.48 | a | 2627.96 | abc | 289.67 | cd | 0.00 | b |

Least square mean value of cell wall bound hydroxycinnamates and 2,4-dihydroxy-7-methoxy-1,4-benzoxazin-3-one (DIMBOA) contents in leaves of maize inbred linesEP39 and EP42 at 2 and 15 days after (dat) infestation with MCB larvae, wounding, wounding + MCB regurgitant application, methyl jasmonate (MeJA) exposure, and no treatment (control).

^a^ *p*CA, p-coumarate; FA, ferulate; DFAT, total diferulates.

^b^ Within each column, different letters indicate significant differences (*p <* 0.05).
